#  Expression of Autophagy Markers Beclin1 and LC3B in Prostatic Carcinoma: An Immunohistochemical Case-Control Study

**DOI:** 10.30699/IJP.2021.530887.2649

**Published:** 2021-09-15

**Authors:** Nanis S. Holah, Marwa M. Serag El-Dien, Shereen F. Mahmoud

**Affiliations:** Department of Pathology, Faculty of Medicine, Menoufia University, Shebin El Kom, Egypt

**Keywords:** Beclin1, Immunohistochemistry, LC3B, Prostatic carcinoma

## Abstract

**Background & Objective:**

Prostatic carcinoma represents the second most common cancer diagnosed in men worldwide after lung cancer and the fourth common male malignancy in Egypt. Autophagy is a natural process that has both oncogenic and tumor-suppressive activities. This study aimed to evaluate the role of Beclin1 and LC3B in prostatic carcinoma.

**Methods:**

This retrospective case-control study was conducted on 110 prostate biopsies divided into three groups (55 prostatic carcinomas, 45 pure benign prostatic hyperplasias (BPH), and 10 BPH with adjacent prostatic carcinoma) retrieved from the archive of the Pathology Department, Faculty of Medicine, Menoufia University, in the period between 2017 and 2020. All biopsies were stained for Beclin1 and LC3B antibodies.

**Results:**

There was a highly significant association between higher Beclin1 and LC3B immunoreactivity score and Gleason score (score 8 and 9) (*P*=0.002 and 0.000, respectively). Moreover, there was a highly significant direct association between Beclin1 and LC3B expression (r=0.52, *P*=0.000). Also, there was a significant stepwise increase in Beclin1 positivity among the three studied groups starting from BPH to prostatic carcinoma passing through cases of BPH with neighboring tumor (*P*=0.000).

**Conclusion:**

From the results obtained in the present study, autophagy markers Beclin1 and LC3B showed upregulation in prostatic carcinoma. Moreover, both were associated with poor prognostic factors. So, it might be necessary to control autophagy flux in prostatic carcinoma. This might be one of the future therapeutic targets for the management of prostatic carcinoma.

## Introduction

Prostatic carcinoma is one of the most prevalent malignant tumors of the male genital system. In 2020, it was the second most common after lung cancer worldwide, accounting for 14.1% of estimated new cancer cases in males with an age-standardized world mortality rate of 7.7 per 100,000 (1, 2)**. **

In Egypt, prostatic carcinoma is the fourth common male malignancy after liver, bladder, and lung cancers, accounting for 7.2% of estimated new cancer cases. Moreover, it is considered the tenth cause of cancer deaths (2.5%) (3).

There were many tumor markers that were used for the diagnosis of prostatic carcinomas, such as prostatic specific antigen (PSA). On the other hand, it might cause misdiagnosis of prostatic carcinoma as it increased with prostatitis after colonoscopy and urinary manipulations as cystoscopy (4, 5).

Autophagy is a natural process responsible for energy metabolism for keeping up homeostasis under stressful conditions (6-8). Autophagy has both tumor suppressive and oncogenic activities (9, 10). On the one hand, it can repress malignant transformation preventing the accumulation of damaged proteins, organelles, and mitochondria. On the other hand, autophagy promotes the survival of cancer cells by providing biochemical reaction substrates derived from the destruction of intracellular organelles and proteins (11, 12). Autophagy may inhibit the initial stage of metastasis by increasing anti-metastatic immunomodulatory factors. Once tumor cells enter blood circulation, autophagy may augment metastasis by protecting the circulating tumor cells from apoptosis (13).

Beclin1 is a scaffold protein that assembles components for promoting or inhibiting autophagy, and its phosphorylation controls autophagy (14). Autophagy regulation by Beclin1 has been shown to play a significant role in tumorigenesis in several cancer types like breast cancer (15, 16). The molecular mechanisms underlying its effects are being elucidated. These studies might lead to important discoveries for Beclin1 targeted therapies in cancer (17).

In mammalian cells, three types of the microtubule-associated protein 1 light chain 3 (LC3) were reported; A, B, and C. LC3B expression being the most valid marker of autophagosome formation and therefore one of the most widely used in situ techniques of autophagy measurement in benign and malignant tissue (18).

The role of autophagy in prostatic carcinoma is controversial and still not completely clarified. It is arguable whether autophagy is activated or inhibited in BPH cells. So, the present study aims to evaluate the immunohistochemical expression of autophagy markers Beclin1 and LC3B in prostatic carcinoma and BPH and their association with the available clinicopathological parameters.

## Material and Methods

This retrospective case-control study was conducted on 110 prostate biopsies divided into two groups: a-55 prostatic carcinoma (10 of them with adjacent BPH), b-55 cases of pure BPH. Formalin fixed paraffin-embedded blocks (FFPE) of those specimens were retrieved from the archive of Pathology Department, Faculty of Medicine, Menoufia University, in the period between 2017 and 2020. Clinical data (age and PSA level) was retrieved from patients' medical files. The studied cases were selected depending on the availability of tissue blocks and patients’ records.

**Histopathologic Evaluation**

From each representative FFPE block, 4 μm thick serial section was cut and stained with hematoxylin and eosin stain for evaluation of the following histopathologic features:

1. Gleason score and grade group according to International Society of Urological Pathology (ISUP) (19)

2. Lymphovascular invasion: present or absent

3. Perineural invasion: present or absent

4. Prostatic intraepithelial neoplasia (PIN): present or absent.

**Tissue Microarray (TMA** **(** **Construction from BPH Cases**

multiple tissue cores with a diameter of 1.5 mm were punched manually from the predefined regions of each BPH donor FFPE block, as a large area of the studied BPH cases better to be represented (20), multiple tissue cores with a diameter of 1.5 mm were punched manually from the predefined regions of each BPH donor FFPE block. We use a tissue arrayer’s needle set provided by the TMA instrument manufacturing company (Breecher Instrument). Worth to be mentioned that we used the TMA needles with a simple handheld holder with great success without the need to use the expensive tissue arrayer instrument (21). 

**Immunohistochemical Staining**

Four μm thick sections from prostatic carcinoma FFPE and BPH TMA blocks were cut and mounted on positively charged slides and analyzed using the immunohistochemical method (streptavidin-biotin amplified system).

 immunohistochemical staining was performed to detect Beclin1 (concentrated rabbit polyclonal antibody, Catalog no. (A7353), ABclonal technology CA, with the dilution of 1:100) and LC3B (concentrated rabbit monoclonal antibody, Catalog no. (A19665), ABclonal technology CA, with the dilution of 1:100). Normal human gastric tissue and normal rat brain tissue were used as positive controls for Beclin1 and LC3B, respectively.

**Interpretation of Beclin1 and LC3B Immun-ostaining Results**

For both markers, when the case showed cytoplasmic brown staining in any number of epithelial cells, it was considered as positive. Both markers were evaluated as positive versus negative. Also, the percentage of stained normal (in BPH) or neoplastic (in carcinoma) epithelial cells was evaluated. Lastly, immunoreactivity score (IRS) was applied: percentage of positive cells was scored as 0, no cells stained; 1, <20% of cells stained; 2, 20–75% of cells stained; and 3, >75% of cells stained. The intensity of immunoreactivity was graded on a scale of 0–3. The IRS equals the product of the scores of percentage and intensity of staining. Negative cases had an IRS of 0, weakly positive cases had an IRS of 1–3, moderately positive cases had an IRS of 4–6 and strongly positive cases had an IRS of >6 (22)**.**

**Statistical Analysis**

The data were collected, tabulated, and statistically analyzed using the statistical package for the social science program for windows version 22 (SPSS Inc., Chicago, IL., USA). Qualitative data were analyzed by Chi-square test (X^2^). Quantitative data were analyzed by applying the Mann-Whitney U test for comparison between 2 groups not normally distributed. Continuous variables were analyzed using two-tailed Pearson’s correlation coefficient (r). A P-value of ≤ 0.05 was considered statistically significant (23)**.**

## Results

**Fig. 1:**
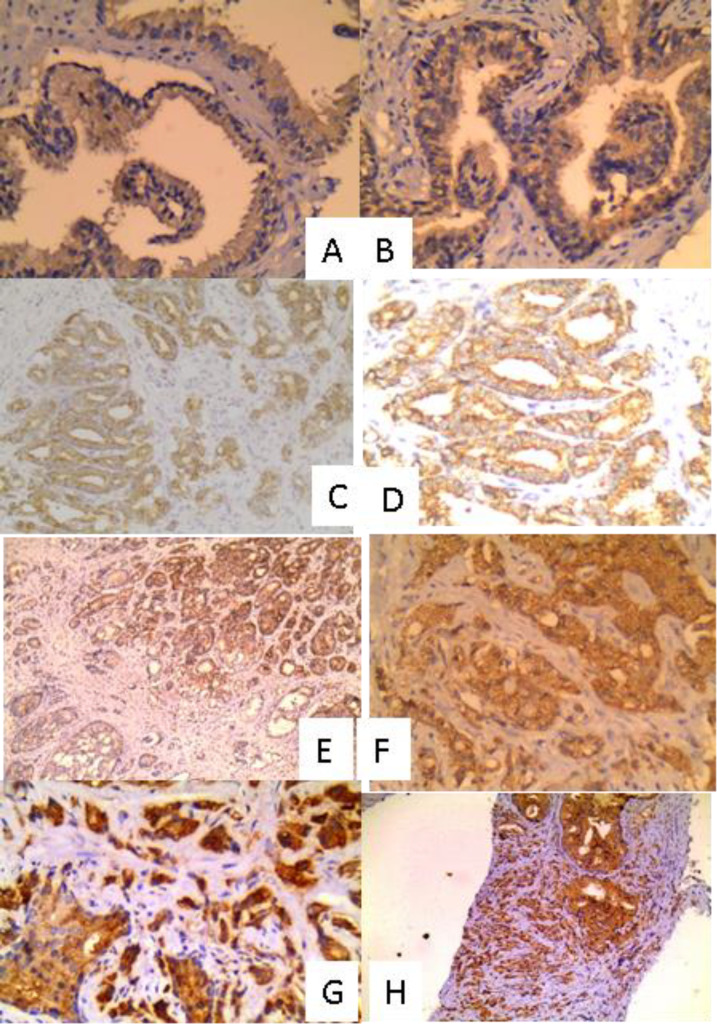
Expression of autophagy markers (Beclin1 and LC3B) in BPH and prostatic carcinoma cases: A: BPH showing mild positivity for LC3B (IHC X 200), B: BPH showing mild positivity for Beclin1 (IHC X 200), C: Prostatic carcinoma Gleason score 6 showing mild positivity for LC3B (IHC X 100), D: Prostatic carcinoma Gleason score 6 showing mild positivity for Beclin1 (IHC X 100), E: Prostatic carcinoma Gleason score 7 showing moderate positivity for LC3B (IHC X 100), F: Prostatic carcinoma Gleason score 7 showing moderate positivity for Beclin1 (IHC X 200), G: Prostatic carcinoma Gleason score 9 showing strong positivity for LC3B (IHC X 200) and H: Prostatic carcinoma Gleason score 9 showing strong positivity for LC3B (IHC X 100)

- Prostatic carcinoma (n=55): the clinicopa-thological were shown in Table 1 (1).
- BPH (55 cases without and 10 cases with adjacent prostatic carcinoma): mean age ± SD (in years) was 67.79 ± 11.87 for cases with adjacent tumor and 65.54 ± 10.77 for cases without. The mean PSA level ± SD (in ng/ml) was 7.13 ± 6.58 for cases without adjacent tumors and 782.52 ± 221.99 for cases with adjacent tumors.
- Results of immunohistochemical expression of Beclin1 and LC3B in the studied three groups of cases (BPH, BPH with adjacent tumor, and prostatic carcinoma cases) were shown in Table 2 and Figure 1.

Association Between Beclin1 and LC3B Expression and Available Clinicopathological Parameters 

**Fig. 2:**
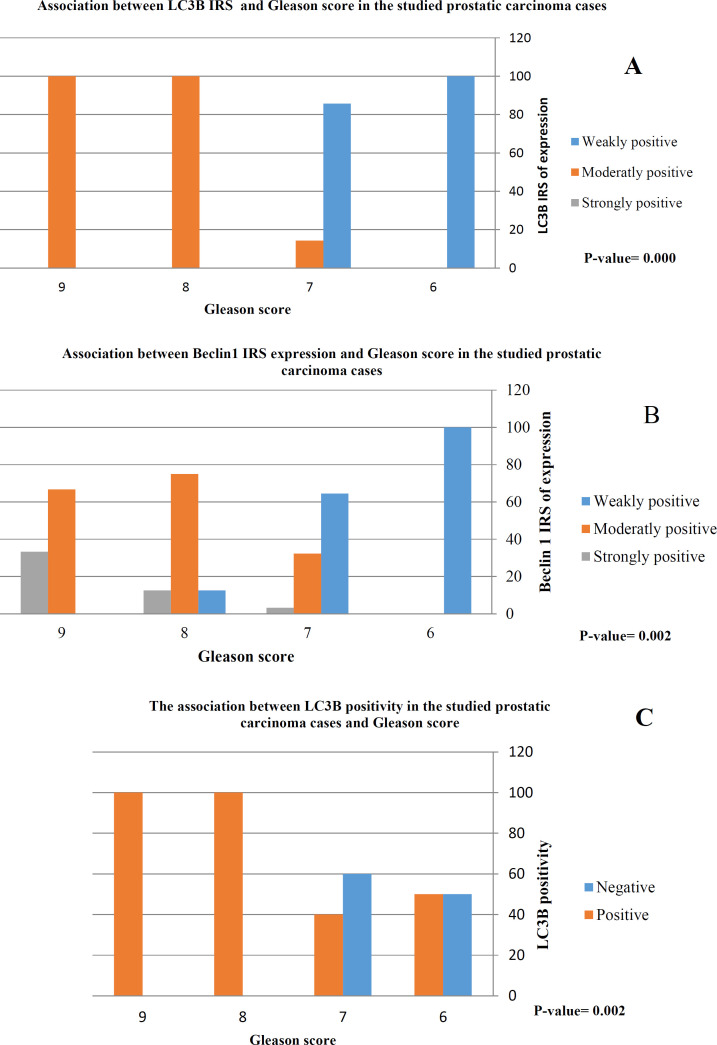
There was a highly significant association between LC3B positivity (C) and moderately and strongly positive expression of LC3B (A) and Beclin1(B) and high Gleason score (score 8 and 9)

LC3B positivity was highly associated with Gleason score (*P*=0.002) and Gleason grade group (*P*= 0.000). All prostatic carcinoma cases with Gleason score 8 or 9 were positive for LC3B. On the contrary, only 50% of prostatic carcinoma cases with a Gleason score of 6 were positive (Figure 2). Moreover, All prostatic carcinoma cases with Gleason grade group 4 or 5 were positive for LC3B. 

Moreover, there was a significant association between Beclin1 and LC3B immunoreactivity score (IRS) and Gleason score (score 8 and 9) (*P*=0.002 and *P*=0.000, respectively) and Gleason grade group (group 4 and 5) (*P*=0.004 and 0.000, respectively). The higher the Beclin1 and LC3B IRS, the higher the Gleason score and grade group.

No significant associations were noticed between studied markers with age and PSA level. Vascular/Perineural invasion and presence of nearby PIN were omitted from comparisons for statistical purposes.

**Table 1:** Clinicopathological characteristics of the prostatic carcinoma cases (n=55).

	No. (%)
Age (years)	
Mean ± SD Median Range	65.73 ± 7.46 64 51-86
PSA level (ng/ml)	
Mean ± SD Median Range	85.82 ± 46.64 74 38-240
Gleason score	
6 7 8 9	6 (10.9%) 35 (63.6%) 8 (14.6%) 6 (10.9%)
Gleason grade group	
1 2 3 4 5	6 (10.9%) 17 (30.9%) 18 (32.8%) 8 (14.5%) 6 (10.9%)
Vascular invasion	
Negative Positive	52 (94.5%) 3 (5.5%)
Perineural invasion	
Negative Positive	51 (92.7%) 4 (7.3%)
PIN	
Negative Positive	53 (96.4%) 2 (3.6%)

No: Number PSA: Prostatic specific antigen PIN: Prostatic intraepithelial neoplasia.

**Table 2:** Immunohistochemical expression of Beclin1 and LC3B in the studied groups

	BPH	BPH adjacent to tumor	Prostatic carcinoma
	n=55	n= 10	n-= 55
	No (%)	No (%)	No (%)
Beclin1 expression			
Negative	31 (60%)	3 (30%)	5 (9.1)
Positive	24 (40%)	7 (70%)	50 (90.9)
Beclin1 percentage			
Mean ± SD	61.67 ± 23.756	78.57 ± 14.634	63.9 ± 22.64
Median	65	75	67.5
Range	20-95	60-95	20-95
Beclin1 IRS			
Negative	31 (56.4)	3 (30)	1 (1.8)
Weakly positive	17 (30.9)	3 (30)	26 (47.3)
Moderately positive	7 (12.7)	4 (40)	20 (36.4)
Strongly positive	0 (0%)	0 (0)	8 (14.5)
LC3B expression			
Negative	31 (56%)	3 (30%)	24 (43.6)
Positive	24 (44%)	7 (70%)	31 (56.4)
LC3B percentage			
Mean ± SD	35.97 ± 15.24	74.29 ± 17.18	35.97 ± 15.24
Median	35	75	35
Range	10-65	50-95	10-65
LC3B IRS			
Negative	31 (56.4)	3 (30)	24 (43.6)
Weakly positive	13 (23.6)	4 (40)	15 (27.3)
Moderately positive	11 (20)	3 (30)	16 (29.1)
Strongly positive	0 (0)	0 (0)	0 (0)

No: Number %: Percent SD: Standard deviation BPH: benign prostatic hyperplasia

**Fig. 3:**
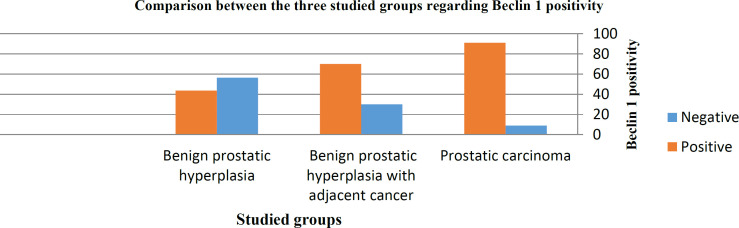
*There is a statistically significant difference between the three studied groups regarding positivity for Beclin1 (*P*=0.000)*

**Comparison Among the Three Studied Groups Regarding Immunohistochemical Results (** Figure 3 **)**

There was a significant stepwise increase of Beclin1 positivity between the three studied groups starting from BPH up to the highest percentage of positive cases appeared in prostatic carcinoma passing through a group of BPH adjacent to tumor. The percentage of cases positive for Beclin1 in the three studied groups was 43.6%, 70%, and 91%, respectively (*P*=0.000).

**Fig. 4:**
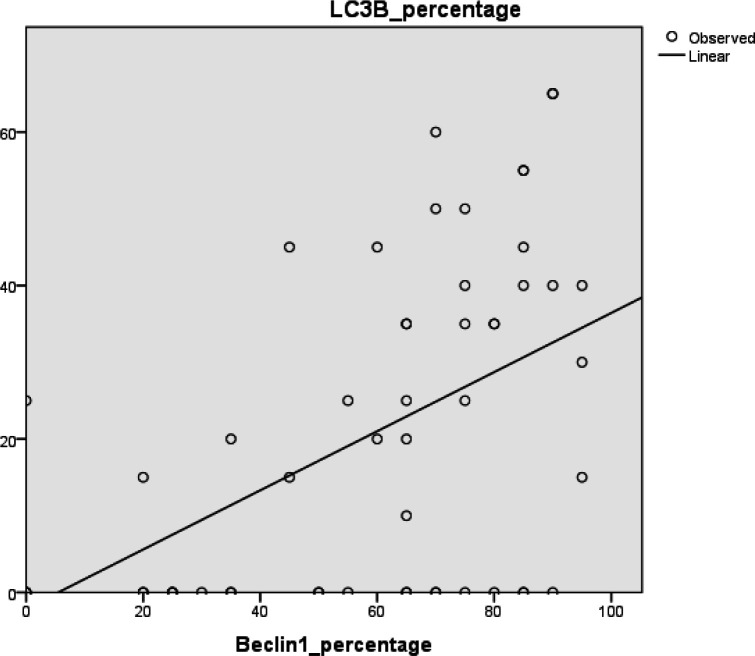
There is a significant linear correlation between the percentage of expression of both Beclin1 and LC3B in the prostatic carcinoma cases (r=0.52, P=0.000)

**Correlation Between Beclin1 and LC3B Percentage of Expression in the Studied Groups of Cases (** Table 3 **) & (** Figure 4 **)**

There was a highly significant direct linear correlation between Beclin1 and LC3B percentage of expression in prostatic carcinoma cases (r=0.52, *P*<0.001).

No significant correlation was noticed between Beclin1 and LC3B expression, in BPH and BPH with nearby carcinoma groups.

 00000000000000

**Table 3:** Linear Correlation between percentage of expression of Beclin1 and LC3B in the studied groups of cases

	BPH	BPH adjacent to tumor	Prostatic carcinoma
Percentage of expression of Beclin1 and LC3B	**r= 0.08**	**r= 0.46**	**r= 0.52**
	***P=*** **0.57**	***P=*** **0.19**	***P=*** **<0.001**

## Discussion

Prostate cancer is one of the leading causes of death in males.Current treatments often lead to the appearance of chemoresistant foci and metastases, with mechanisms still partially unknown (24). There is a controversial and still the role of autophagy in prostatic carcinoma is not completely clarified. So, the present study aimed at evaluation the immunohistochemical expression of autophagy markers Beclin1 and LC3B in prostatic carcinoma and BPH and their association with the available clinicopathological parameters.

The present study demonstrated that 50/55 (90.9%) of prostatic carcinoma cases showed Beclin1 positivity and this was close to the study performed by Baspinar* et al.* (2014) (25), who found positive beclin1 expression in 84.9% of prostatic carcinoma cases. And these results were in contrast to Liu* et al.* (2013) (22), who demonstrated downregulation of Beclin1 in prostatic carcinoma and this contravery in te results might be due to the difference in the number of the studied prostatic carcinoma cases as they use 34 cases only and also the difference in the used technique as they used western blotting assay.

Regarding LC3B, the present study found that 56.4% of prostatic carcinoma cases showed LC3B positivity, and this disagreed with Falasca *et al.* (2015) (26), who demonstrated* an *88% increase in the level of LC3B protein in prostatic carcinoma cases.

Regarding the expression of the used autophagy markers in BPH in the present study, Beclin1 was expressed in 40% of cases, and LC3B was expressed in 44% of cases, and these results were close to that of Liu* et al.* (2013) (22) who found that Beclin-1 was expressed in 34.15% of BPH cases and LC3 was expressed in 36.59% of cases. And also close to that of Oh* et al.* (2020) (27), who found LC3B expression in 50% of BPH cases. 

The current study demonstrated a highly significant association between increased autophagy in the form of positive LC3B expression and high Gleason score (score 9) and high Gleason group (group 5). And also in the form of moderately and strongly positive Beclin1 and LC3B expression and high Gleason score (score 9) and high Gleason group (group 5) and these results were in accordance with other studies that explained that as there is a “dual-faced” role of autophagy either tumor suppressor or tumor promotor according to the stage of the malignancy (24, 28). At later stages of tumor progression, autophagy is induced as a protective mechanism to allow cancer cells in the central areas of the tumor to survive in the local low-nutrient and low-oxygen conditions (29)**.**

There was a stepwise pattern of Beclin1 positivity regarding the three studied groups starting from prostatic carcinoma group as 91.7% of cases showed positive expression to the BPH group as 43.6% of cases showed positive expression passing through BPH adjacent to the tumor group as 70% of cases showed positive expression as this comes in concordance with (27) who found that autophagy was significantly decreased in BPH cell lines compared with cell lines of normal prostate and this might be associated with the etiology and progression of BPH (27) so the current study might suggest a role for Beclin1 in initiation and progression of prostatic carcinoma**.**

These results might be explained as decreased autophagy might result in inhibiting autophagic cell death and/or promoting cell survival, leading to increased proliferation of tissue cells that results in the development of BPH (27). Also, autophagy protected cancer cells against damage from a low nutrient supply, ionizing radiation, and chemotherapy (30), promoting cancer progression**. **But these results disagreed with Mathew* et al.*, 2007 (31), who demonstrated Beclin1 acted as a tumor suppressor, not a promotor, as it suppressed tumor progression by limiting chromosomal instability**.**

A previous study demonstrated that inhibition of autophagy might increase the response of prostatic carcinoma cells to the other therapeutic modalities as autophagy inhibition enhances apoptosis induced by 5-FU (22)**.** Another study reported that after receiving a low dose of radiotherapy, the phenomenon of autophagic vacuole accumulation could be observed in the cells of prostatic carcinoma, colon, and breast cancer. Autophagic vacuoles as a defense mechanism might protect cells against radiation. Suppose we could inhibit the formation of autophagic vacuoles by autophagy inhibitors. In that case, the mortality rate of cells receiving radiation might be higher (32) So, it might be necessary to control autophagy flux in prostatic carcinoma**.**

Small number of radical prostatectomy specimens was a limitation to this study 

## Conclusion

Autophagy markers Beclin1 and LC3B showed upregulation in prostatic carcinoma. Moreover, both were associated with poor prognostic factors. So, it might be necessary to control autophagy flux in prostatic carcinoma. This might be one of the future therapeutic targets in managing prostatic carcinoma.

## Conflict of Interest

The author(s) declared that they have no potential conflicts of interest concerning the research, authorship, and/or publication of this article.
